# Effects of Abiotic Stress on Soil Microbiome

**DOI:** 10.3390/ijms22169036

**Published:** 2021-08-21

**Authors:** Nur Sabrina Natasha Abdul Rahman, Nur Wahida Abdul Hamid, Kalaivani Nadarajah

**Affiliations:** Department of Biological Sciences and Biotechnology, Faculty of Science and Technology, Universiti Kebangsaan Malaysia, Bangi 43600, Malaysia; nursabrinatasha@gmail.com (N.S.N.A.R.); nurwahida9827@gmail.com (N.W.A.H.)

**Keywords:** abiotic stress, soil microbiome, microbial population, rhizosphere, beneficial microbes

## Abstract

Rhizospheric organisms have a unique manner of existence since many factors can influence the shape of the microbiome. As we all know, harnessing the interaction between soil microbes and plants is critical for sustainable agriculture and ecosystems. We can achieve sustainable agricultural practice by incorporating plant-microbiome interaction as a positive technology. The contribution of this interaction has piqued the interest of experts, who plan to do more research using beneficial microorganism in order to accomplish this vision. Plants engage in a wide range of interrelationship with soil microorganism, spanning the entire spectrum of ecological potential which can be mutualistic, commensal, neutral, exploitative, or competitive. Mutualistic microorganism found in plant-associated microbial communities assist their host in a number of ways. Many studies have demonstrated that the soil microbiome may provide significant advantages to the host plant. However, various soil conditions (pH, temperature, oxygen, physics-chemistry and moisture), soil environments (drought, submergence, metal toxicity and salinity), plant types/genotype, and agricultural practices may result in distinct microbial composition and characteristics, as well as its mechanism to promote plant development and defence against all these stressors. In this paper, we provide an in-depth overview of how the above factors are able to affect the soil microbial structure and communities and change above and below ground interactions. Future prospects will also be discussed.

## 1. Introduction

Increase of greenhouse gases (GHGs) like carbon dioxide (CO_2_), methane (CH_4_) and nitrous oxide (N_2_O) has led to global warming, which directly influences the world’s climate. World food security is affected by climate change. Climate change can affect precipitation patterns and increase global temperature which can directly affect the agriculture system. According to IPCC [1], food prices are expected to go higher in 2050 as climate change has significant effects on crop yield through increase of CO_2,_ which will affect the temperature and crop productivity. For example, C3 crops received benefits from high CO_2_ levels as it can increase photosynthetic rates and result in more carbohydrates and higher sugar content in crop development [2]. However, elevated CO_2_ will also negatively impact plants by reducing nutritional values of crop since the increase of C/N ratio helps plants build tolerance against soil-borne pathogen, and may also trade-off on crop nutritional quality and productivity [2]. Further, changes in precipitation patterns will affect the water availability/water level. Heavy precipitation can cause crop damage, soil erosion, and flooding, while low precipitation may result to drought which can negatively impact agricultural productivity. Drought, or a lack of sufficient water, is one of the most prevalent stresses that impacts crop development, yield production, and quality. It is projected to worsen as the world’s population grows [3,4]. On the other hand, water-logging can lead to anaerobic conditions which will reduce oxygen (O_2_) levels in soil due to water filling the spaces that typically allows for gas exchange between the atmosphere, soil, and soil microorganism resulting in considerable reduction in gaseous diffusion [4]. This condition will affect cellular respiration and notably change the biochemical and physiological processes in plants. Voesenek et al. [5] and Tamang and Fukau [6] reported that during flooding, ethylene increases and the level of Zn, Mn, Fe and S is increased to toxic levels. Both of these abiotic stresses have the potential to affect crop yield in severe conditions, and as a result, both remain as indicators for global food security [7]. 

Maintaining high crop yield is important to maintain profit and to supply food for the world’s population. Crop yield is influenced by type of soil, agricultural practices, disease virulence and mitigation, climatic effects, which include UV radiation, temperature, humidity and precipitation rate. All these factors interact and influence the health and yield of crops. Plant growth-promoting organisms have been gaining attention due to their potential to improve the development of plants in harsh environments. They use various mechanisms to stimulate the root development and improve the absorption of water. These organisms that can be found in the rhizosphere can help plants reduce stress and improve the absorption of water. They also produce plant hormones that can help reduce drought tolerance. Various organisms that act on the surface of plants by carrying out various actions such as carbon sequestration, nitrogen fixing, P solubilization, and soil remediation are known to have various mechanisms of action. Colonization by beneficial rhizospheric microorganisms will enhance the interaction between plant growth-promoting organism and host plant which will aid in plant growth and development by providing beneficial micronutrients and macronutrients to the plants [8]. In addition, these microorganisms play a vital role and influence the development of plant organs above-ground. 

Plant biomass production is directly influenced by soil microbial biodiversity and symbiotic relationship between microorganisms in the soil which contributes to plant nutrient uptake and other physiological processes. Different farming traditions such as type of fertilizer used, crop rotation and tillage also play a major role in the microbial community. Abiotic stressors such as drought, extreme salinity, and other abiotic stressors influence plant carbon metabolism to varying degrees, depending on the stress rate, plant species, and plant tissue type. Duenas et al. [9], Mueller and Bohannan [10], and Wang et al. [11], consistently reported that utilization of N fertilizer in wheat does not affect arbuscular mycorrhizal fungi (AMF) significantly as plants release more exudates with long term N fertilization hence modified soil properties following substantial N addition. AMF receives a higher proportion of plant-derived ^13^C which modifies the stability of AMF diversity. High N application will contribute to low soil pH. N and P have a higher effect on AMF species diversity as the addition of these sources reduces plant carbon availability to AMF and has the potential to shift AMF from mutualism to parasitism, reducing rhizosphere AMF diversity [11]. Shift in soil microbial communities such as bacteria and fungi will affect the biogeochemical cycles in the soil thus affect the nutrient assimilation and plant defense mechanism. In terms of abiotic factors, bacterial communities in soil are dependent on soil properties like pH, carbon, nitrogen, and moisture content [12,13,14,15]. However, fungal communities are governed more by biotic factors such as plant diversity [16]. Bacteria and fungal community have a similar effect towards soil variables such as pH, carbon, nitrogen, and moisture content, but the regional abiotic factor such as climate exerts a bigger impact on variation in bacterial community than fungal communities [16,17]. In this review, we discuss how abiotic stressors affect soil microbe communities and how microbes adapt to the different abiotic stresses.

## 2. Importance of Soil Microbiome

Rhizosphere is the region around roots where root exudates play a prominent role in controlling communications between soil microbiome. Soil microbiome are largely made up of bacteria and fungi and they have roles to play in keeping the soil healthy and fertile. The root microbiome’s bacterial and fungal members are known to have either commensal, pathogenic, or beneficial relationships with their hosts as well as each other. Soil microbiomes are key in biogeochemical cycles such as nitrogen, carbon, sulphur and phosphorus. In the nitrogen cycle, *Rhizobium* sp. are known to symbiotically fix nitrogen in the legumes in exchange for fixed carbon and sugar. Free living bacteria that can fix nitrogen is the *Azotobacter*. Microbes involved in the C cycling are Proteobacteria, and AMF, which are obligatory symbionts that rely on host plant for carbon, and the ectomycorrhizal fungi (ECM), which are symbionts with the ability to mineralize organic carbon. In the sulphur cycle, the anaerobic, phototrophic sulphide-oxidizing purple and green sulfur bacteria, as well as certain anoxygenic facultative cyanobacteria, can construct sulphur cycles with sulphide-forming bacteria by producing elemental sulphur and sulphate. AMF enhance the sulphur uptake in the soil [12]. In alkaline soil, P precipitates are easily accessible [13] and phosphate solubilizing microorganisms (PSMs), solubilize insoluble organic and inorganic phosphorus compounds to easily assimilated form. PSM include a number of well-known strains of genera *Rhizobium*, *Pseudomonas* and *Bacillus*, as well as genera *Aspergillus* and *Penicillium*, AMF and actinomycetes [14]. Soil microbiome are also involved in other biochemical reactions such as, antibiotic production for pathogen defense, biomass decomposition, biodegradation, maintenance of soil structure, nutrient uptake and stress tolerance in the soil. A few potential bacteria and fungi have been studied to help boost development of agricultural plants such as tomato, wheat and paddy. Further details are as in Table 1.

### 2.1. Bacteria

Proteobacteria can grow and adapt well to soil with low carbon sources, making this the most abundant bacterial group in the soil [15]. Acidobacteria, the second largest ubiquitous group in soil plays an essential role in the soil C cycle and has the ability to breakdown cellulose and lignin [15]. Actinobacteria can be found in disease suppressive soil and promotes plant growth and root nodulation which has a symbiotic interaction with N_2_-fixing bacteria [12,29]. N_2_-fixing bacteria also known as diazotrophs, are commonly found in plant rhizosphere and are a substantial source of nitrogen in soil [15]. Diazotrophs can access N via N_2_-fixation, which uses nitrogenase enzyme systems to convert dinitrogen to ammonium. They are key contributors to the biosphere’s nitrogen economy, accounting for 30–50 percent of total nitrogen in crop fields [29,30]. Symbiotic interactions between plants, actinobacteria, and mycorrhizal fungi enable shrubs and trees to adapt to dry, flooded, polluted, and saline environments [15]. 

Rhizobiaceae is one of the well-known plant growth promoting rhizobacteria (PGPR) that form symbiotic relationship with legumes for N_2_-fixation in soil [4]. These growth-promoting rhizobacteria can be categorized as (i) rhizhospheric, (ii) rhizoplane, (iii) endophytic and (iv) specific structural bacteria based on their connection with roots [31]. Direct mechanism of PGPR include biofertilization, root stimulation, rhizoremediation, and plant stress control, while antibiosis, induction of systemic resistance and competition for nutrients are the indirect mechanisms of PGPR [32]. It has been shown that, inoculation of beneficial bacteria are able to reduce the use of fertilizer and is a cost effective initiative in reducing the use of agrochemicals [33,34,35]. PGPR such as *Burkholderia* sp. and *Pseudomonas* sp. assist P solubilization and nutrient uptake in rice (*Oryza sativa*) and oil palm (*Elaeis guineensis*) [36]. Furthermore, inoculating rice with N_2_-fixing bacteria and P-solubilizing bacteria improves leaf chlorophyll content, plant nutrient absorption, and yield, and reduces N_2_ and P fertilizer consumption by 50% [32]. In the absence of pathogen, rhizobacteria produce hormones such as gibberellic acid (GA), indole acetic acid (IAA), ethylene (ET) and cytokinins (CK), which helps in plant development. CK regulates cell division, main root growth, nodulation, and branching, whereas GA promotes shoot development, cell elongation, and seed germination [31,37]. In rhizobacteria, both tryptophan-dependent and tryptophan-independent pathways have been found as leading factor to IAA production, which will regulate cell division, elongation, and differentiation [37]. When ET rates are elevated, the plants experiences stress, and root development is hampered. However, enzyme 1-aminocyclopropane- 1-carboxylate (ACC) deaminase produced by bacteria breaks down the plant ethylene precursor ACC into ammonia and ketobutyrate, resulting in low ethylene levels [37]. 

Rhizospheric and endophytic bacteria promote suppression of pathogens, and improves mineral availability through plant hormones. For example, inoculation of *Beijerinckia* spp. resulted in substantial nitrogen content in several maize hybrids [30]. *Gluconacetobacter* can synthesize phytohormones IAA and GA type A1 and A3 which affects the development of plant roots while gluconic acid produced is used to promote chelation in P and Zn solubilization [38]. Inoculation of *G. diazotrophicus* is used widely as biological control of other pathogenic microorganisms such as *Xanthomonas* sp., *Colletotrichum* sp. and *Fusarium* sp. which are causative agents that result in diseases worldwide by stimulating genes which control ET pathway in the plant defense system [38].

Actinobacteria have the ability to suppress the dissemination of a number of plant pathogens including *Erwinia amylovora,* which causes apple fireblight and *Agrobacterium tumefaciens,* which causes crown gall disease [39]. It also helps in soil activities including ammonium fixation, breakdown of cellular tissue, and synthesis and decomposition of humus [38]. Antibiotics, vitamins, amino acids, and other physiologically active compounds can be manufactured by actinobacteria, including IAA, which affects several basic cellular functions such as cell division, elongation, and differentiation. [38]. Aside from that, by producing hydrolytic enzymes, actinobacteria play an important role in the recycling of organic materials in the environment by producing hydrolytic enzymes which specialize in the decomposition of refractory and indecomposable organic materials such as cellulose and lignin. This will result in a lot of dark black to brown pigments, which will add to the soil’s dark colour.

Archaea has a prospective function in nutrient recycling, particularly carbon, nitrogen, and sulphur. Archaea biogenically produces and oxidizes methane (CH_4_), an essential hydrocarbon and energy source, essential for carbon absorption and organic matter mineralization [15]. Cultivated Crenarchaeota and Euryarchaeota, for example, thrive autotrophically and play an important role in carbon absorption from bicarbonate (HCO_3_) or CO_2_. Denitrification of soil by ammonia-oxidizing archaea (AOA) oxidizes nitrite to nitrate. Thaumarchaeota is the largest contributor to ammonium oxidation and one of the most abundant in the planet and easily found in the soil [40,41,42]. Thaumarchaeota is known to live in a wide range of environmental conditions. They live in fresh water to oceans, from pH 3.5 to pH 8.7, and from low temperature environment such as Artic to very high temperature environments (between 74 °C to 124 °C) such as hot springs [41]. They receive ammonia from urea and cyanate [42]. However, nitrification process by AOA may also lead to nitrate leaching from soils, causing groundwater and surface contamination with nitrous oxide (N_2_0), resulting in further acceleration of global warming [15].

### 2.2. Fungi

Fungi can live in a wide range of environmental conditions as they have high plasticity and capacity. Fungi are famous as decomposer in soil, and can produce a variety of extracellular enzymes which help to convert organic matter to CO_2_. Fungi also can help to reduce metal toxicity in soil by absorbing heavy metals such as Cd, Cu, Hg and Pb [15]. Other than that, fungi also are known as biological controllers which helps to control diseases caused by phytopathogenic fungi. For example, *Aureobasidium pullulans* is a yeast-like fungus that has potential to control disease caused by fungal pathogens in apple, strawberry and grape, *Rhizofagus irregularis* and *Talaromyces assiutensis* can be used to control disease in olives, and *Trichoderma harzianum* is a well-studied fungus that can be used to control many diseases including *Fusarium* wilt, root rot and bacterial wilt [43,44,45]. Fungal communities in soil are important in P solubilization and N_2_ uptake for host [46,47]. Mycorrhizal fungi are important in nutrient uptake, where the symbiotic relationship helps increase water and nutrient uptake efficiencies in olive plants. It also helps improve protection towards biotic and abiotic stress [15]. The appearance of AMF colonizing plant roots favour different crops undergoing *Verticillium* attacks. AMF can boost micronutrient absorption and tolerance to a variety of abiotic stresses [48,49,50]. Most of AMF are from sub-phylum Glomeromycotina and phylum Mucoromycota [51]. They take up products from photosynthesis and lipids as obligatory biotrophs in order to complete their life cycle [52]. AMF-mediated growth protects plants from fungal infections, as well as enabling water and nutrient absorption from adjacent soil [53]. As a matter of fact, AMF are important endosymbionts that contribute to plant production and ecological function [53]. Vesicular arbuscular mycorrhizal (VAM) on the other hand are fungi that establish symbiotic relationship in the roots of host plants and can be utilized to improve rate of phosphate absorption from the soil and increase phosphorus level which is essential for plant growth [53,54,55]. VAM fungus produces and releases organic compounds (siderophores) that enhances P desorption in P pool labile soil [56]. VAM can use organic acids to dissolve insoluble and low-soluble P sources which is a component of the soil mineral crystalline structure [56].

## 3. Factors That Affect Soil Microbiome

Microbial populations have a key influence in soil fertility and health. Any external stress such as drought, submergence and chemicals will alter the chemistry and physics of the soil and hence affect its biology. Soil physicochemical properties influence gaseous adsorption between environment, soil particles and soil microorganisms. It also shows a significant affect towards soil pH. Soil physical chemical properties include soil porosity, soil pH and soil organic carbon. These properties interlink with each other to influence the microbial density and activities of soil microbiome. Although microbial populations are influenced by soil physical chemistry, soil physical chemistry is greatly influenced by abiotic factors such as climate change and biotic factors such as parent material, agricultural practices, and land use. Studies of the last decade have revealed how microorganisms in soil can affect the growth of plants and affect crop yield. Understanding soil microbiome is very difficult as the microbiome is very sensitive to abiotic stresses such as soil pH, salinity, UV radiation, temperature and rainfall resulting in fluctuating profiles. Having more information on soil microbiome related to abiotic stress may help find a new and effective solutions in navigating losses due to environmental stresses.

### 3.1. Soil pH

The most important factor that can affect the population of soil microbiomes is soil pH which can be influenced by metal toxicity, soil structure and texture, source of water and land use intensification. Beneficial soil microorganisms and plants favor a pH range of 6 to 7, thus changing in soil acidity or alkalinity are frequently followed by changes in the microbial composition and activity [57]. Research done by Zhang et al. [58] contradicts reports by Rousk et al. [59] where pH shows a significant effect towards fungal community. Other research by Vasco-Palacios et al. [60], in different forest soil show a variation of fungal community related to the forest type, soil pH and soil carbon content. Generally, fungal communities may be affected by the soil pH but other environmental and edaphic factor such as soil physico-chemical properties also plays a huge impact on the structure and dynamics of soil fungal community. Fernandez-Calvino and Baath [61], reported that a slight change in pH will lower the original community of the soil microbes and allow the growth of adapted bacterial community. Besides, excessive land use can increase soil pH which will cause carbon concentration and water retention in soil to decline, hence, alter the soil structure [62]. Research done by Malik et al. [56] states that excess land use will increase soil pH but soil microbial community will be affected differently depending on the type of soil [62]. Acidic soil pH shows low diversity of diazotroph communities around alpine meadow soils, which suggests that low soil pH can reduce N_2_-fixing process in acidic soils [29]. Different types of bacteria have different tolerance towards soil acidity and alkalinity. For example, *Azospirillum* density is not affected by soil pH but *Bradyrhizobium* communities can live well in acidic soil while *Mesorhizobium* communities will be reduced at low pH soil [29]. Alkali soils have comparatively low amounts of soil organic biomass and nutritional content, and hence are incapable of sustaining agricultural development. The poor performance of alkali soils is largely due to reduced microbial activity. Jones et al., [63] and Mayerhofer et al., [64] discovered that different Acidobacteria subgroups have different pH sensitivities, where acidobacterial subgroups 4, 6, 7, 9, 16, 18 and 25 were associated to alkaline environments while subgroups 2, 6 and 13 was commonly associated to acidic environment. 

### 3.2. Soil Temperature

Fluctuations of climate affects the global temperature; CO_2_ level and precipitation patterns. Microorganisms can be classified into three groups which are mesophiles where their optimal growth temperatures range from approximately 20 °C to 45 °C, psychrophiles where the microorganisms live in cold environment and the optimal growth temperature ranges from 15 °C (or lower) to 20 °C and thermophiles which have higher optimum growth temperature ranging from around 50 °C to higher [65]. Soil microbes in temperate forests show a significant relative abundance where temperature increase of 5 °C, results in higher bacterial population than fungi [66]. Different temperatures will affect the key enzymes that can be found in N_2_-fixing bacteria. For example, at temperatures around 5 °C, vanadium nitrogenase is the most effective enzyme used for N_2_-fixation process. At warmer temperatures, around 30 °C, molybdenum nitrogenase is more effective due to higher affinity for N_2_ compared to vanadium nitrogenase. Higher temperature also may favour the growth of new pathogenic strains at higher latitude [2]. As reported by Goicoechea [2], development of disease caused by *Verticilium dahliae* in olive cultivar is determined by soil temperature, where the increase in CO_2_ will favour the salicylic acid pathway and suppress the jasmonate acid pathway which is essential for a stronger defence against *V. dahliae* attack [2]. Soil temperature also affects AMF colonization differently at different regions with different C:N ratio. Studies by Frater et al. [67], show that AMF colonization increased parallel to increase temperature and pH where the experiment took place at Central United State but Goicoechea [2] mentioned that AMF colonization across the Mediterranean decreased when the temperature increased. Jerbi et al. [68] agreed with Frater et al. [67] where increase in temperature appears to help AMF with better root colonization by elevate plant root elongation while at colder temperature, nutrient acquisition by AMF is reduced leading to a decrease in mycorrhizal colonization. Therefore, different AMF have different optimum temperature for growth and development. 

### 3.3. Soil Aeration

Soil hypoxia is a condition where the soil has less oxygen mainly caused by waterlogging [69]. Hypoxia causes stomatal closure in plant tissues, which leads to energy shortages and disrupts the growth of plant roots, reducing their capacity to absorb water and inorganic nutrients [70]. Even in aerobic species, oxygen inhibits nitrogenase irreversibly. As a result, diazotrophs must use defence mechanisms to keep N_2_-fixation going in the presence of oxygen. This involves avoiding oxygen by growth approach, isolating nitrogenase from oxygen spatially and/or temporally, and using biofilms as hindrance to oxygen diffusion [29]. Diazotrophs can also remove oxygen by boosting substrate consumption, which boosts respiration rates and lowers oxygen levels [29]. Switchgrass has been found to increase microbial development in the rhizosphere via exudation and, as a result, substrate consumption. This process is presumably observed in the switchgrass rhizosphere. To compensate for the loss of oxygen, diazotrophs increase their respiration. However, if carbohydrate supply is sufficient, diazotrophs can still fix N_2_ even under high oxygen pressure. N_2_-fixation, with a modest energy advantage over assimilatory nitrate reduction, can actually be an energetically advantageous process for NH_3_ acquisition under optimum oxygen conditions. Actinobacteria are mostly aerobic, which means they require oxygen for metabolism, which is why they were hardly seen in flood plains [39].

### 3.4. Soil Physico-Chemical Properties

Soil texture is an important component which is connected to more complicated soil properties including the primary features of the water holding capacity, cation-exchange capacity, and hydraulic conductivity [71]. The texture of soil is associated with the various morphologies and the locality of chemicals found on the surface due to the surface absorption of mineral particles [72]. It is composed of silt, sand and clay particles, which has a substantial impact on the composition and biomass of soil bacteria [72]. Soil texture may impact the way diazotrophs control oxygen as a result from the connection between texture and substrate (i.e., C) and oxygen diffusion. Increased clay concentration in soils can generate microaerophilic and anaerobic microsites where bacteria are shielded from oxygen, therefore sustaining bigger populations and/or more effective N_2_ fixers [29]. Loss of water may lead to soil compaction in certain types of soils and this can directly impact the density, diversity and activity of soil microbes. Compaction is a kind of soil deterioration that involves the disruption of soil structure and a reduction in pore sizes. It is more common and severe in clay soils, and can be worsened through the use of machinery in fields [2]. Clay soil supports N_2_-fixation better than sand with more nitrogenase activity [73]. High soil compaction will limit the development of the fungal hyphae [2]. Marupakula et al. [25] reported that the depth has significant effect on the density of fungi in soil where the total percentage of fungal Operational Taxonomic Unit (OTUs) dropped, with significantly positive reductions between the O (organic) and E (eluviated) horizons [74]. O horizon is the uppermost stratum of soil horizon and comprises of live and decayed elements such as plants, leaves and rotted animal carcasses. The humus fertilizes the soil and provides nutrients for the developing plants. Therefore, a great abundance of microbial population can be found because of the availability of nutrients in this horizon. E horizon is a mineral horizon containing mainly silicates which are not beneficial to the microbes present. N-fertilization increased density of fungi at the organic horizon but does not affect mineral horizon and illuvial horizon, but N fertilizers show negative impact to the number of fungal OTUs associated with roots, where the OTUs declined significantly at all parts of soil horizon. The effects of nitrogen on fungi in the roots were significant, and most indicators in all horizons fell considerably [74]. 

### 3.5. Soil Moisture

According to Siebielec et al. [75], moisture has a greater impact on respiration than temperature. Microbial communities in damp soils are functionally diversified, however, excessive soil moisture, on the other hand, may result in decreased microbe biomass [75], owing to oxygen conditions that are inhospitable to aerobic bacteria, including Gram-negative, Gram-positive, and mycorrhizal fungi [53,76]. The moisture content of soil is an extremely important component that affects soil biological activity [77]. Excess water in the soil environment is especially dangerous to aerobic microorganisms [78] as availability of O_2_ is considerably lower in water compared to air [79]. Microbial growth and activity is inhibited [75], mineralization of N and C was reduced [80], and structure of microbial communities is shifted [81] in water. 

Cells store enough water to maintain its metabolism and turgidity by keeping the cytoplasm at a greater osmotic potential (more negative) than the outside environment [82]. Soil microorganisms may collect organic and inorganic substances when the water content is low (high water potential), increasing the osmotic potential inside cells. The importance of moisture on soil microbiota will be discussed under the drought and submergence sections. 

## 4. Soil Microbiome under Abiotic Stresses

### 4.1. Drought

Drought is a significant impediment to agricultural productivity. Drought is currently the climate phenomenon that holds the biggest negative impact on food security. The severity and frequency of drought is expected to increase over the next decade. Furthermore, drought season has a pronounced effect on the soil microbiome, as moisture and temperature [15] are determinant effectors of microbial growth and activity as mentioned in our previous section. The moisture level influences soil microbiota and causes shifts in microbial activity and structural diversity [83,84]. Whereas, a rise in temperature due to drought season has detrimental impact on microbial biomass [84] and microbial population abundance [85]. The combination of high temperature and water deficits can restructure soil microbial communities more broadly. Increased evapotranspiration caused by drought may lower soil water supply below a stress threshold, causing microbial activity to be suppressed [86]. Aside from that, drought-induced reductions in labile carbon and nitrogen entering the rhizosphere might be a contributing factor in the loss of microbial phyla such as Verrucomicrobia, Proteobacteria and Acidobacteria which are heterotrophs [87] and sensitive to nitrogen ratios [88]. 

Further as a consequence of drought, the respiration of microbes is decreased by about 30% at low moisture, and growth productivity estimates based on C immobilization vs. net mineralization of nitrogen is differed, indicating disturbance of cellular activities in microbes [89]. According to Meisner et al. [90], drought-induced warming can reduce the abundance of 16S rRNA genes in soil microorganisms on a seasonal basis. In a variety of situations, drought has also been associated to an increase in monoderm bacteria in the roots of several plant species [91]. A number of studies across several plant species [91,92] have indicated that the microbiome of plant roots changes during drought, favoring Actinobacteria and many other Gram-positive species, which substitute the Gram-negative taxa that are predominantly present [91]. Wipf [93] discovered that the relative abundance of Actinobacteria surged in the root microbiome of *Sorghum bicolor* both during drought and more gradually as temperature increased. Other than Actinobacteria, and Gram-positive bacteria, Firmicutes were also found in abundance during drought stress [94]. Actinobacteria and Firmicutes produce exospore and endospores that are resilient to desiccation thus thrive well in drought [95,96]. 

Study by Barnard [97] found that dryness triggered ribosomal synthesis in Actinobacteria, which could explain their increased abundance following drought [93]. Apart from that, Acidobacteria also showed increased numbers after drought. However, Acidobacteria’s response to stress varies and is dependent on soil pH [94]. According to the findings, Acidobacteria are found in relative abundance in acidic soils (3.0–6.5 pH) as lower pH promotes their abundance, and their abundance declines in less acidic soils [98]. According to Ward et al. [99], since Acidobacteria thrive in acidic soils, they are capable of surviving drought better at low pH than other bacterial phyla. Besides bacteria, AMF, particularly Glomeromycota can increase during drought stress [100] and they may impart drought resistance to host plant by increasing activities of antioxidant enzymes, that curb oxidative pressure and encourage better water consumption and biomass production. Glomeraceae family may exhibit their opportunistic behavior by spending most of their energy on producing more descendants [101] and developing characteristics that make it possible to thrive in dry climates [102]. Study by Chodak et al. [94] found that Planctomycetes are probably one of the only Gram-negative phylum that is able to survive in drought stress. This might be due to their unique characteristics of cell walls with no peptidoglycan, differentiation into multiple compartments by inner membranes, and generally larger genomes [103]. The decrease in Gram-negative bacteria may also be caused by the detrimental impact of drought and rewetting on C-cycling in soils [94].

Soil microorganisms use a variety of techniques to deal with drought stress and maintain their survival. First, microbes can withstand drought if they release compatibleosmolytes as protective mechanism that work in concert with plant-secreted osmolytes [104]. Microbes will limit their intercellular osmotic potential through production of solutes such as amino acid osmolytes (glutamine, glutamic acid, proline, taurine) which can synthesize cellular proteins, in order to maintain water when soils are parched and water potential drops [105]. Osmolytes are produced when bacteria and plant ecosystems are exposed to abiotic stressors. Osmolytes maintain protein structural integrity by scavenging reactive oxygen species (ROS) from various organelles and preventing cellular damage [106] from oxidative stress [107].

Next, microbes preferentially collect organic compounds that diminish solute potential without interfering with cellular metabolism, such as glutamate, glycine, betaine, proline and trehalose [89]. These solutes maintain the pressure of cellular hydration and turgor by keeping an osmotic balance without damaging the cytoplasm’s osmotic potential [108,109]. Compatible solutes are organic compounds with a low molecular mass that does not engage negatively with macromolecules [109]. However, microbes’ capacity to adopt physiological acclimatization mechanisms, such as producing suitable solutes, which cost energy and demand carbon, will be reduced as water stress grows [89]. If dryness limits microbial growth by limiting substrate availability and reducing diffusion, extracellular polymetric substance (EPS) synthesis should preferentially increase as an effective drought adaptation method [110]. According to More et al. [111] microbes can increase their function and survival in hostile environments by improving their local habitat. EPS is one means of doing so where it predominantly contains polysaccharides, protein, and DNA produced by living and dying cells. Even at low matric potential, EPS acts like sponge, slowing the drying process thus retaining water by allowing action at low matric potential [112]. Fungi are reported to be more abundant and less impacted by drought because their hyphal development is widespread and exploratory. Moisture fluctuations result in shifts in the make-up of microbial communities on lower trophic level favouring the fungal community as fungi perform relatively better over bacteria in dry conditions [113]. Bouskill et al. [114] reported that antibiotics were produced at much higher rates during drought stress as a physical reaction to competing for scarce resources with other bacteria or as triggers for drought-response mechanism such as biofilm formation [114]. The increased availability of antibiotics during a drought could be the result of rapid environmental changes thus leading to formation of bacteria with drought resistant traits that have been initially stagnant or scarce [114].

### 4.2. Submergence

Flooding causes the soil to be compacted with water which in turn restricts gas exchange between the atmosphere, soil, and microorganisms. Hence, this results in significant reduction of O_2_ concentration in soil [4]. Other than that, flooding is known to influence the distribution of soil microbial community by changing soil pH and nutrient status. Flooding increases ethylene accumulation within the plant organ due to the limited outward gas dispersion underwater. Certain soil microbes may affect plant phenotype by interfering with the ethylene levels by producing enzyme which can degrade the ethylene, hence reduce ethylene levels in plant [115]. Flooding, according to previous studies, lowers fungal communities in soil, including fungal pathogens, by providing unfavourable conditions for fungal communities while favouring anaerobic bacteria and therefore boosting anaerobic bacterial communities. [80,116]. In the event of flooding, soil microbe populations favours anaerobic microbes while obligate aerobic organisms will gradually decrease. Flooding also causes the bacteria from the water to be transferred to the soil. Other than that, Furtak et al. [117], reported that anaerobic bacteria such as *Anaeromyxobacter* and *Malikia* may only be present after flooding events while obligate aerobic bacteria such as Xanthomonadaceae, completely disappeared as a result of flooding. Alphaproteobacteria, Betaproteobacteria and Deltaproteobacteria are dominant populations in agricultural soil and can survive submergence. Betaproteobacteria which are closely affiliated with *Aquaspirillum* sp. have a few members that can grow anaerobically with nitrate and some that can catabolize ethanol [118]. Soil microbial communities show measurable changes 7 days after flooding and takes between 21–24 days to reach a stable community which shows significant difference in communities between flood and non-flooded soil.

Research done by Sánchez-Rodríguez et al. [119] have the same results with Bossio and Scow [120] and Bai et al. [116] where Gram-negative bacteria decreased under flooding with fresh water and Gram-positive bacteria increased. Studies done by Bal and Adhya [48] shows that inoculation of seeds with Gram-positive plant growth promoting rhizobacteria (*Bacillus* sp., *Microbacterium* sp., *Methylophaga* sp., and *Paenibacillus* sp.) helps rice variety IR42 to withstand submergence stress by reducing the inhibitory effect of ethylene stress. Although Unger et al. [76] stated that flooding reduced fungal communities, Sánchez-Rodríguez et al. [119] reported that AMF density was increased in the event of flood in saline water. Several studies showed that density of AMF community increased with low precipitation which reduced soil humidity and elevated O_2_ concentration [68]. Other studies by Silvana et al. [121] and de Oliveira et al. [122] reported higher precipitation can enhance AMF colonization. These contradictions in results can be due to differences in soil temperature, soil texture, soil pH and host plant. 

When submerged in fresh water, water molecules will diffuse freely into the microorganism and increase the turgor pressure of the cytoplasmic membrane and eventually lead to cell lysis [30]. To cope with this stress, bacteria have evolved numerous ways to enable them to grow in a wide variety of solute concentrations, such as adjusting their intracellular osmolarity or enhancing cell wall stability. First, the bacteria will activate aquaporins which help to control the diffusion of water and other small molecule into the cell. Next, in the event of temporary or short-term osmotic stress, the expression of the potassium transporter such as Kup, KdpFABC and TrKA will be regulated intracellularly. Third, under prolonged osmotic pressure, the proVWX-encoded ABC transporter will allow bacteria to take in the osmoprotectants glycine betaine and proline from the environment or manufacture glycine betaine from the extracellular precursor choline [32]. In addition, to avoid cell lysis during sudden osmotic pressure, mechano-sensitive channels such as MscL and MscS that can be found in many bacteria, archaea and fungi will act like valves which open pores of the cell membrane to a larger diameter thus releasing osmotically active ions and solutes from the cytoplasm to stabilize cell [29]. These channels appear to sense tension inside the membrane rather than pressure across it as their means to discharge excess cell turgor pressure [39]. 

### 4.3. Metal Toxicity

Anthropogenic interventions and excessive agrochemical application results in negative impact on soil microbial communities and functional diversity, leading to deterioration of soil health, raising concerns regarding the impact of intensive land use practices and excessive pesticide usage on human and environmental health. Soil physicochemical qualities are a major leading force for change in soil microbiome communities, and it is well known that heavy metals have a profound impact on microbiome populations. Metals are mainly released to the environment through natural weathering from metal-rich bedrock. Other than that, human activities, such as industry, mining, fertilizer production and wastewater disposal contribute to metal content in soil. Excess fertilization, for example, has severe environmental consequences such as, increased GHGs, and phosphorus run-off, which can increase possibility of eutrophication occurrence [123,124,125]. While organisms need metals such as zinc (Zn), iron (Fe) and manganese (Mn) in small amounts to enhance growth, development and metabolism, some heavy metals such as cadmium (Cd), lead (Pb), chromium (Cr) and mercury (Hg) found in the environment may disrupt life cycle of living organisms by causing cell membrane damage, disrupting enzymatic and cellular processes, and resulting in DNA structural damage [126]. For example, it has been reported that Zn-added broth results in deformation of *Gluconacetobacter* *diazotrophicus*, causing pleomorphic, aggregate-like cells that can impede the Zn chelation process [38].

Metals such as chromium, vanadium, arsenic and selenium are used by soil microbes in metabolism process as electron donors or acceptors. These metals are used in considerable amounts without causing harm to soil microbes [126]. Microorganisms in soils showing lower levels of heavy metal-contamination were discovered to use more carbon for assimilation, with less CO_2_ emitted during the dissimilation process, than microorganisms in contaminated soils [126]. Microorganisms that live in heavy metal-contaminated soils, on the other hand, require more energy to thrive in unfavourable circumstances and will generate more CO_2_ during the dissimilation process which contributes to increase of global temperatures [126]. However, research done by Ma et al. [127], shows that soil microbial alteration by heavy metal pollution in mangrove wetland restricts CO_2_ production while promoting CH_4_ fluxes. These contradictory results may be related to the abundance of archaebacteria which can live in harsh environments like oceans [41]. Microbes that have been exposed to heavy metals for an extended length of time will progressively develop tolerance, which will be critical in the restoration of contaminated ecosystems [128].

Firmicutes, Proteobacteria, and Actinobacteria have been observed to predominately colonize heavy metal polluted locations, whereas AMF frequently colonized nutrient poor soils polluted with heavy metals [129,130]. Seneviratne et al. [131] reported that a diverse range of bacteria and fungi create organic acids as natural heavy metal chelating agents. Fomina et al. [132] reported that *Beauveria caledonica* released oxalic and citric acid that was capable of solubilizing Cd, Cu, Pb, and Zn. The filamentous hyphal structure of AMF helps to penetrate deep into the soil and provide advantage in adsorbing heavy metals [133]. Other than that, root exudates that contain carbohydrates, amino acids and flavonoids can stimulate microbial activity in rhizosphere. In the rhizodegradation process, plants release certain enzymes such as oxygenase and dehalogenase which are capable of degrading organic contaminants in soils and creating nutrient rich environments for soil microbes hence, increasing rhizospheric microorganism growth and activities [134]. Further, the increase of metabolic activities of microbes helps to enhance degradation of metal pollutants around the rhizosphere.

Cadmium, a common metal found in the soil, interrupts soil microorganisms’ enzyme activities such as denaturing enzymes, deteriorates membrane structure, followed by interruption of function and interference with enzyme synthesis in cells [135,136]. Apart from that, pH has a key influence in cadmium availability in soil, where higher pH, clay and organic matter content in the soil lowers Cd availability through reducing metal mobility in the soil [137,138]. Due to its poor mobility and weaker affinity for soil colloids, Cd has been demonstrated to be more lethal on enzymes compared to Pb [128]. In addition, heavy metals have the potential to disrupt microbial reproduction and induce morphological and physiological abnormalities. Therefore, hazardous heavy metals in the environment may impact biodegradation processes [139] and result in deleterious effects to the environment. 

### 4.4. Salinity

One of the most serious soil degradation problems facing the world today is salinization. In agriculture, the usage of agricultural inputs can lead to salinity. Salinity may be caused by the use of sewage sludge and manure, as well as municipal garden waste products resulting in salt accumulation in soil when frequently applied [140]. Due to high osmotic pressures as well as harmful ions and imbalance in nutrition [141], salinity causes suboptimal plant development and reduces activity of soil microbes. This is attributed to the fact that salinity changes water relation of plant tissues, nutrition and ion imbalance, and toxicity owing to the accumulation of Cl^-^ and Na^+^ ion levels in the plant tissues and soil [142,143,144,145]. The salt content in soil (salinity) can influence soil processes, and defines the osmotic pressure, and the sodium content in the soil’s exchange complex (sodicity), which further regulates systemic stability of the soil [146]. Sodicity would develop gradually from salinity. Soluble salts lower the ground water solute potential (make it harsher), pulling water off from cells and causing plasmolysis which potentially kills microorganisms and roots [146]. An increase in salinity leads to a theory known as “rapid osmotic phase” where osmotic stress causes water removal from the soil in matter of minutes, which is followed by “slower ion toxicity phase” or “hyperosmotic stress phase”. This is described by a high concentration of toxic ions, slowing cell division and growth rate, resulting in a challenging environment for roots and microorganisms [147]. 

There are two type of salinity tolerant microbes: halophiles, which live in high salinity environments and require salt for growth, and halotolerant organisms, that can adapt to saline environments. According to Mainka et al., [148] the salt tolerance of halophilic bacteria is characterized as follows: (i) halotolerant, which can grow in saline surrounding, however do not need high salinity to grow, (ii) weak halophiles (1–3% of NaCI), intermediate halophile (3–15% of NaCI) and intense halophile (15–30% NaCI). These bacteria frequently have novel enzymes with polyextremophilic features that act amid salinity conditions, such as cellulases, xylanases, proteases, amylases, lipase and galatinase [149,150]. These halozymes have salt-tolerance or salt-dependent catalytic properties [151]. Halozymes have the same enzymatic properties as non-halophilic predecessors, however, their structural features differ significantly, allowing them to function in extreme conditions [151]. This includes a significant proportioning of aminoacids on the surface of proteins and the need for a high salt concentration for efficient biological processes [151]. Owing to the massive conglomeration of partially hydrophobic groups and protein surface hydration caused by carboxylic group found in glutamate and aspartate, these halophilic enzymes are secure in the involvement of high salt concentration [151]. Halophile-produced enzymes can be significant biological molecules, such as phytohormones and exopolysaccharides which are crucial in plant-microbiome interaction and also aids in the stability of the soil structures and water-holding of soil particles [152]. These are also useful towards bioremediation of such pollutants in saline settings [153,154]. Salt-tolerant plants have vast beneficial microbiomes in their rhizospheres that enables the plants to grow and cope against drought and extreme salinity [155,156]. 

**Figure 1:**
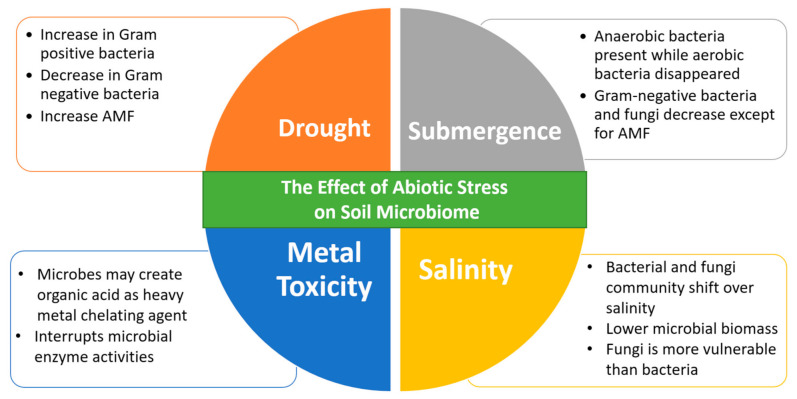
The above diagram shows the effect of changes in environmental factors on the soil microbial composition, health and well-being.

Microorganisms and plants may accumulate osmolytes to adapt to low osmotic pressure. Unfortunately, through complex biosynthesis pathway, osmolyte production costs a considerable amount of energy and involves a massive C-skeleton [157] resulting in diminished activity and growth. Many findings proved that salinity lowers microbial activity, relative abundance along with altering the shape of microbial communities [108,150,158]. Study by Andronov et al. [159] also found that the taxonomic composition of bacterial and fungal communities shifts over salinity gradients. This study further demonstrates that the makeup of the microbial community altered with salinity, where certain microbial OTUs from the non-saline source soil, which was employed as the original substrate, were filtered out or became less numerous as salinity increased [159]. It is presumed that salinity lowers the biomass of microbes as well as their activity and community structure due to their large area volume ratio, high permeability of cell membrane and rapid turnover rate, mostly due to osmotic pressure causing cells to shrink resulting in water efflux from the microbial cells and therefore retarding their growth [160,161]. When it comes to salt stress, fungi are more vulnerable than bacteria [108,162,163], hence in saline soils, the bacterium/fungi ratio might be raised. Salinity resistance varies among microorganisms causing alterations in composition of microbial communities [164], resulting in the notion that salinity can be a critical indicator of microbial diversity and composition at the community level [158]. The primary organic osmolytes are proline and glycine betaine, while the main prevalent inorganic substances employed as osmolytes in salt tolerant bacteria are potassium ions [165]. Nevertheless, as previously stated, synthesizing osmolytes that are organic needs a significant amount of energy. Since the formation of osmolytes from inorganic salts is likely to be hazardous, so only exclusive halophytic microorganisms that have developed salt-tolerant enzymes can thrive in very saline conditions [146]. Figure 1 below shows how the four parameters addressed here affect the microbial population in the soil.

## 5. Link between Soil Microbiome and Plant Genotype

### 5.1. Plant Genotype

Breeding and domestication processes are believed to have a substantial influence on shaping the rhizospheric microbiome and may interfere with the interactions of beneficial microbes and plant [166,167]. According to Pérez-Jaramillo [162] plant genotype has a small impact on the microbiome composition of the rhizosphere, albeit this varies depending on the soil and plant species studied. Plant architecture has changed dramatically as a result of domestication and plant breeding. Although modifications of the aerial portions are much more evident, root morphologies are certainly altered as well, albeit selection for drought resistance, flood tolerance, or yield qualities may all have direct or indirect influences on the plant structure. Microbial communities linked with roots would most likely be affected by such changes [166]. Peiffer et al.’s [163] maize study found that genetic varieties in maize had a minor but substantial influence on alpha and beta proteobacterial diversity in the field, based on a detailed study of 27 inbreed lines. Similar findings in maize were discovered in a study by Szoboszlay et al. [168], which looked at rhizosphere activities in wild and cultivated varieties of corn and showed that plant genotype had a small impact on the rhizosphere. Knief et al. [169] identified rice rhizosphere and phyllosphere functions, while Edwards et al. [170] found rhizosphere variations across cultivars *of Oryza sativa* japonica and indica subspecies, as well as domesticated *Oryza glaberrima* (African rice). Edward et al. [170] also made an observation that rice genotypes had a considerable impact on the makeup of the rice root microbiome when cultivated under controlled greenhouse settings, but no impact was seen when cultivated in the wild. Bulgarelli et al. [171] looked at associated bacteria in root of a commercial variety, a locally-adapted variety, as well as a wild accession of barley, and discovered a tiny but substantial influence on microbial communities around the plant genotype’s roots. Ellouze et al. [172] discovered different chickpea genotypes were linked to varying bacterial biomass as well as differing fungal populations and diversity. Depending on the plant growth stage, different potato cultivars have been demonstrated to change the total bacterial and Beta proteobacterial communities [173]. 

Genotype effects were also discovered in the soil rhizosphere bacterial population linked to two distinct soybean cultivars [174]. Although changes in microbial composition at the genotype level appeared to be minor, genes involved in immunological, nutritional, and stress responses might modify the abundance of certain microbial consortia, which would have a significant impact on host performance [175,176,177,178]. Haney et al. [179] described one example of this shift, where the genotype variations in wild Arabidopsis accessions had the tendency to interact with *Pseudomonas fluorescens* and build on host fitness. Furthermore, there have also been studies that revealed even minor variations in genotypes of the plant could still exert a significant impact on microbial rhizosphere. Bressan et al., [180] discovered that the microbial population of transgenic Arabidopsis root is shifted due to the exogenous production of glucosinate. Likewise, Cotta et al. [181] discovered that modified genotype of maize encoded distinct *Cry1F* and *Cry1Ab* Bt toxin genes which affects the species richness of archaeal and ammonia-oxidizing bacterial communities within that rhizosphere. Apart from that, plants such as rice (*Oryza sativa*), corn (*Zea mays*), barley and wheat (*Triticum aestivum*) have special tissues known as aerenchyma which can help the plant adapt to submergence stress, where this specialized tissue helps in O_2_ transfer [182,183,184]. The aerenchyma transports oxygen to the roots via two pathways which is either through respiration process or through radial oxygen loss (ROL) where O_2_ is transferred from the roots to the rhizosphere in this system [4]. This will allow ROL to develop aerobic conditions in the rhizosphere, where it potentially alters the rhizosphere communities by promoting the growth of aerobic bacteria [4]. The rhizosphere, particularly the rhizoplane and endosphere makeup, can be influenced by the immune system of the plant and their root exudates, which then affects the root architecture and microhabitat [166].

The microbial rhizosphere community composition is influenced by deposits and secretions, which results in the microbial activities impacting plant development and health. Microbial communities differ depending on the host plant’s genotype due to variable exudates as well as plant physiology and development. The recruitment of the root microbiome is most directly linked to root exudates. Carbon-based compound such as mucilage, organic acids, enzymes, amino acids, sugar and ions make up root exudates [185]. These are released either indirectly (root cells lysis/senescing roots) or directly via a cycle reputed as “rhizodeposition”. Exudates from the roots are believed to ‘prime’ the soil around the roots ecosystem, such that, they would recruit favorable bacteria to the rhizosphere, resulting in higher rhizosphere respiration rates and bacterial biomass percentage compared to surface soils [186]. Microorganism may respire between 64–86% of plant rhizodeposits [173,174,187,188]. Micallef et al., [189], also reported that different accessions of Arabidopsis have unique exudate profiles, and thus, their bacterial communities in rhizosphere are diverse among these accessions [189]. Consequently, the production of distinct root exudates in same species of plant by various genotypes has been shown to have a major influence in selecting “rhizospheric allies” [190]. 

### 5.2. Leguminous and Non-Leguminous Plant

The main distinction between legumes and non-leguminous plant is that, leguminous plant associate symbiotically with N_2_-fixing bacteria from the genus *Rhizobium* (Alpha-proteobacteria) [187,191], whereas non-leguminous plants interact in a symbiotic way with N_2_-fixing bacteria from the genus *Frankia* (Actinobacteria) [188,192]. The ability to colonize the rhizosphere and interact with plants is possessed by a large number of N_2_-fixing bacteria from various bacterial phyla [193]. The root nodules developed by legumes and non-legumes plants is one of the most specialized and effective in N_2_-fixing processes. Rhizobia are Gram-negative significant nodule-forming bacteria found in the alpha-proteobacterial genera that engage in endosymbiotic relationships with family Fabaceae. They constitute genera such as *Allorhizobium*, *Bradyrhizobium*, *Rhizobium*, *Methylobacterium*, *Phyllobacterium*, *Ochrobactrum*, *Devosia*, *Mesorhizobium*, *Agrobacterium*, *Sinorhizobium*, *Azorhizobium*, *Pararhizobium*, *Neorhizobium*, and *Aminobacter* [194,195]. The genera *Cupriavidus* and *Burkholderia* classified as beta-proteobacterial were also involved in N_2_-fixing [196]. 

Actinobacteria, like *Frankia* sp. can associate with a wide range of plants that are related to eight different families of non-legumes, generally known as actinorhizal plants, [197] such as Myricaceae, Casuariaceae, Betulaceae, Coriaceae, Datiscaceae, Rhamnaceae, Eleagenaceae and Rosaceae [198,199]. Instead of just fixing nitrogen symbiotically, *Frankia* can also fix nitrogen aerobically by forming vesicles, which are not reported in rhizobia [200]. The unicellular paraphyletic rhizobia and filamentous *Frankia* sp. are phylogenetically separated, implying that the two main species of N_2_-fixing symbiotic bacteria have inherited N_2_-fixing mechanism through divergent evolutionary origins [201]. The expected benefit of the relationship for the host plants in all of these associations and symbioses is the fixed-nitrogen from the symbiotic partner, who will in return obtain reduced carbon and essential resources from the host plants [194,202]. The N_2_-fixing bacteria associated to plant roots may offer the necessary mechanisms to shield the nitrogenase complex from oxygen exposure i.e., the oxygen demand of the respiratory system in the nodule is satisfied adequately by simultaneously protecting nitrogenase from O_2_ [196,203]. According to Scheublin et al., [204] legumes potentially establish a three-way symbiotic relationship with *Rhizobium*, as well as AMF, the phosphorus-acquirer. This study discovered that AMF colonies differed between legumes and non-legumes. Moreover, legumes have a far higher prevalence of this trait than the non-legumes, implying that AMF are specialized for plants with rich concentrations of nitrogen [204]. Dawson [205] shares this viewpoint, but with regard to non-leguminous plants, where the Actinorhizal plants can also form myccorhizal interactions, and these three-part symbioses (*Frankia*-host plant-mycorhiza) offers them the aptitude to grow in poor and marginal soils. 

Rhizospheric microbial populations are influenced by a variety of biotic and abiotic factors. The types of plant and also genotype of the plant, howbeit, is found to be crucial in determining the ultimate form of the rhizospheric microbiome [206,207]. The host plant’s identity has a major impact on the profile of its microbiome as different plant species grown next to one another might have different microbiomes [208]. The plant immunity and exudates of the roots could have caused this, since root outflux varies by plant type and physiological stages, as they improve the rhizosphere by increasing the nutrient bioavailability and stimulate plant growth by altering the physical, chemical and microbiological activities [209] and therefore it is possible that the fungal and bacterial species composition was altered depending on these differences in the rhizosphere [210]. For instance, the relative abundance of native rhizobacteria is shown to be regulated by the host plant [190,211]. Similarly, under some stressful situations, plants produce various metabolites in their root exudates [212], such as organic acids like citric, malic, fumaric and succinic acids, and these aid the host plant in attracting particular microbial species [213]. On the whole, the composition of rhizospheric organisms and their abundance is strongly dependent on plant hosts’ genotypes and plant types.

## 6. Soil Microbiome under Different Agricultural Practices

Agricultural methods are believed to have an impact on the soil ecosystem, which in turn determines the soil’s microbiology and plant’s growth rate. Agricultural yield and productivity are increased through a variety of strategies. Depending on their practices and components applied; agricultural management is classified as either conventional or agro-ecological. Agro-ecological farming strives to tread a fine line between human and natural interest, whereas conventional farming is technologically disassociated from nature. In other words, agro-ecological practice is an agricultural system that uses ecologically based pest controls and biological fertilizers derived primarily from animal and plant wastes, as well as N_2_-fixing cover crops to reduce environmental effect as well as to increase soil nutrient content. Conventional farming on the other hand depends heavily on the utilization of high input modern agriculture that employs chemical pesticides and synthetic fertilizers [214]. Considering that the microbiome is involved in nearly every soil activity, and microbial composition, diversity, abundance, and activity will primarily enhance crop health and long-term productivity of agricultural land [215]. Thus, it is critical to maintain the microbiome in outstanding shape in order to preserve the long-term viability of the land. To that end, it is critical to understand the impact of different agricultural practices on soil microbiome, so that safer, more appropriate agricultural practices can be implemented. 

Agro-ecological systems have shown a resurgence in microbial activity and biomass from the quality and quantity of manure [216]. Araujo [217] and Sayara et al. [218] stated that through the use of natural amendments like straw and compost, soil biochemical and microbiological processes were enhanced, and the microbial biomass was increased and might be sustained for extended length of time. Study by Lori [219] discovered that organic or agro-ecological farming showed up to 84% higher microbial biomass of carbon and nitrogen and greater accumulation of phospholipid fatty acids, protease, urease and dehydrogenase compared to conventional farming. Microbial growth and activity in the soil increased rapidly when readily decomposable organic matter like glucose was added. As a result, a substantial concentration of readily decomposable organic matter in the soil might promote rapid microbial development, leading to an increase in microbial activity and biomass in the soil. 

Melero et al. [220] demonstrated that organic farming resulted in much higher microbial biomass than conventional management, owing to a substantial supply of accessible C. This study correlates with another study by Sardiana [221] where when organic and conventional plots were compared, the result indicated that organic plots had greater microbial biomass. The significant difference of microbial biomass between the conventional and organic farming system reveal the continuous influence of organic C input on the amount of microbial biomass in organic farming [221]. Larsen et al. [222] observed that any reduction in yields observed in organic farming was most likely due to greater weed pressure due to no tillage. Ratio of carbon and nitrogen (C/N) can also regulate the dynamics of soil microbial biomass. The C and N contents of soil microbial biomass are mostly lower in conventional practices compared to organic practices, suggesting that there may be significant disturbances in the microbial biomass of conventional practice and that the persistent intake of organic waste with rich C/N ratio resulted in an increase of microbial biomass [217,223]. Further, Esperschütz et al. [224] found higher phospholipid fatty acid (PFLA) levels in organic farming, which indicates changes in microbial community and composition.

When it comes to providing sustainable soil-based ecosystem services, microbial community structure is just as essential as microbial community abundance and activity. A vast and dynamic microbial community with little functional diversity may struggle to adapt to changing climatic circumstances, but a diversified population may be more resilient to environmental changes [219]. Numerous studies indicate that microbial abundance and diversity were higher in organically managed soils than in conventionally managed soils [216,225]. Reduced tillage [226], cover cropping [227], and organic fertilizer and compost manure application [228] may boost microbial diversity by enhancing organic carbon and nutrient supply in the soil, resulting in increase of heterotrophic microbiota [229]. The use of organic amendments and strategies related to reducing or eliminating chemical inputs, as well as biological plant protection, are all linked to increased microbial diversity in organic systems [230]. Increase in diversity of microbes enhances metabolic activities and heterogeneous species abundance, implying a steady and functioning redundant population, contributing to potent ecosystem performance based on robust trophic ecosystem [231,232]. Hartmans et al. [225] postulated based on dispersion, evenness, and richness, that increased availability of a beneficial substrate such as farmyard manure improved richness by favoring copiotrophic species, whose dominance decreased evenness. In the absence of farmyard manure, however, the ecosystem became less eutrophic, with a more diverse distribution of nutrients, lowering richness and enhancing dispersion and evenness, probably by promoting certain oligotrophic species that proliferate slowly [225]. 

In conventional farming, the decline in microbial diversity can be explained by the usage of plant-protection products such as insecticides, herbicides and fungicides that directly or indirectly cause long-term impact on the soil microbiota and ecosystem services such as nutrient cycling, fixation and suppression of disease [233,234,235]. These agrochemicals decrease the diversity of soil microbiome due to their ability to block or remove particular species of microorganisms that are incapable of proliferating in the conventional agricultural environment [236]. According to Olden et al. [237] the decreased heterogeneity (i.e., lower beta diversity) of the microbial population in conventional farming shows evidence of ecological homogenization, which is a way of developing community homogeneity across a variety of functional and taxonomic groups. Poor agricultural practices such as rigid monocultures of crop, fertilization, and excessive usage of agrochemicals results in a chain reaction of biodiversity losses, microbiota homogeneity and altered functional gene pool. These series of events disrupt ecosystem balance and causes decline in ecological resilience [238,239]. The changes in organic and conventional farming systems are intricate and therefore would require further study and elucidation for concrete conclusions to be made. 

## 7. Agricultural Inputs and Soil Microbiome

Agricultural inputs include organic fertilizers, inorganic fertilizers, plant protection like pesticides, and water. Each of these items are used to increase production and profit [240,241]. Over time, greater implementation of fertilizer and pesticides, rise in irrigation and cultivation, as well as major landform conversions, have all contributed to increase in agricultural output. However, heavy application of fertilizers and pesticides in agriculture can be damaging to the ecosystem and human health. Metal toxicity is widespread in fields and farms as a consequence of chemical fertilizers and pesticides [242,243,244,245]. The impact on the soil microbiota, on the other hand, is complicated and varied. 

### 7.1. Fertilizer

Soil microbes are fertilization-sensitive, and their reactions to manure and/or mineral fertilizers in soils has received considerable attention globally [225,246]. Fertilization affects soil microbial diversity by altering the nutrient concentration of the soil [247]. Fertilizer application affects the development of disease, alters the bacterial community composition and function [248,249,250,251], and contributes towards the formation of soil microbial biomass [252]. In agriculture, farmers may use either organic and mineral fertilizers, depending on their needs. Organic fertilizers include (i) manures (livestock feces), (ii) compostable organics, (iii) beneficial microbial inoculants, and (iv) humic compounds [253,254]. Since these are utilized in agro-ecological farming, organic fertilizers provide a variety of C compounds with varying chemical compositions, ranging from labile to recalcitrant, that soil microbes can employ to boost their growth rates and biomass throughout the mineralization process [255]. Microbial proliferation in the soil is also promoted when raw and composted organics like kitchen waste are added [256]. Study by Poll et al. [257] proves that the addition of manure boosted microbial biomass and activity of xylanase and invertase compared to long term annual application of farmyard manure. In addition, organic manures also boosted microbial variety, and C turnover [250,258]. Meanwhile, humic compounds can directly increase microbial activity by providing carbon substrate, nutritional supplementation, and improved nutrient absorption through cell membranes [259]. Increased levels of humic acid produced from brown coal or compost boosted growth of aerobic bacteria, with no impact on filamentous fungi and little effect on actinobacteria [259,260,261]. If humic acid is the only carbon source in soil, microbial activity may be hindered [262]. 

Microbial inoculants particularly plant growth promoting microorganism (PGPM) is one of the most efficient soil bacteria for promoting plant growth and development [215,263]. PGPM can benefits plants in three mechanisms: (i) Biofertilizer (i.e., N_2_-fixing bacteria and phosphate-solubilizing bacteria) to aid in plant nutrient absorption by delivering fixed nitrogen or other nutrients [264]; (ii) Phytostimulators that can stimulate plant growth by creating plant hormones [264,265,266]; and (iii) Biological control agents to protect from phythopathogenic organisms and abiotic stress [264]. However, positive microbial inoculants may transiently affect microbial biomass [267], and the rise in activity or biomass may be triggered by native inhabitants preying on the freshly introduced microbes [268]. If the organisms that are newly introduced are not well acclimatized to the soil types as the existing population, the changes in population may be restricted to only the inoculation season [269]. 

Mineral fertilizers, sometimes called chemical fertilizers, are not entirely made of natural resources and it is a quick approach to provide plants with essential macro- and micronutrients. As a result, environmental contamination has grown dramatically. So, managing the use of chemical fertilizers is crucial in order to fulfill crop nutrient requirements, while minimizing the risk of environmental harm. Chemical fertilization can alter the activity of microbial enzymes in the soil, as well as the pH and structure of the soil [270,271]. Persistent usage of mineral fertilizers can lower pH of the soil, which has been linked to decreased microbiome diversity and significant shifts in the makeup of microbial community [258]. According to Singh and Gupta [272], long-term nitrogen fertilizer application or in combination with other mineral fertilizers, alters the nitrogen cycle and associated bacterial population. A reduction in the soil organism’s activity following inorganic fertilization might be attributed to the toxic effects of metal pollutants including mineral fertilizers which also contributes to soil acidification and compaction [273]. Generally, K and N fertilizers have extremely low degree of pollutants, but P fertilizers frequently have high quantities of lead, mercury and cadmium [274,275]. In the presence of these hazardous heavy metals, enzymes may be inactivated, and cell function may be damaged due to metals forming chelation and precipitates with important metabolites [276]. Long-term and short-term pollution of heavy metals in soil leads to negative impact on microbial activity, particularly the microbial respiration and soil enzyme activity which results in a drop in microbial population [277,278]. It also resulted in reduction in genetic diversity in the population, relative to untreated or uncontaminated soils [276]. Further, greater microbial activity is observed when plants and animals are growing together [279]. 

There are also circumstances where organic and mineral fertilizers are used jointly. Overall, mineral fertilizers have varying impacts on soil microbes, whereas organic fertilizers were shown to have beneficial longstanding impacts. Good microbial profile was found in the treatment of organic fertilizer and balanced mixture of mineral fertilizer and organic manure [280] compared to mineral fertilizer singularly. A combination of organic and mineral fertilizers may be beneficial to the crops since they may compensate for each other’s inadequacies. Study by Zhong et al., [280] discovered that the PLFA levels of bacterial, actinobacterial and Gram-negative bacteria were cumulatively higher in treatment of organic manure combined with NPK fertilizer. This indicates that the mixture of organic manure and mineral fertilizer stimulates soil organism development whereas mineral N fertilizer inhibits it. Generally, fertilizer treatment decreased microbial biomass around pH5 but showed increase as pH rose. However, overall, Geisseler and Scow [252] discovered that fertilizer application increased microbial biomass in soil about 15.1% compared to non-fertilized soils. 

Aside from organic and synthetic fertilizers, the agricultural industry is today supported by the presence of new technologies, namely nano fertilizers. Nanotechnology offers the ability to create slow-release, high-efficiency nanofertilizers [281] that enhance soil nutrients, create healthier soil ecosystem, improve microbial communities [282] and thereby lowering cultivation expenses. Study by Rajput [283] showed that the population of soil microbial communities exposed to nanofertilizer was much higher than in soil amended with chemical fertilizer. The study on green pepper plants by Nibin et al. [284] found that the slow-release of nanofertilizer treatment positively impacted the microbial population and enzyme activity of the soil. In this study the soil treatment of nano NPK (12.5 kg ha^−1^) and the foliar spray of nano NPK (0.4 %) had highest bacterial population and also higher urease, acid phosphatase and dehydrogenase activities [284]. It is presumed that due to the slow release in nano-fertilization, a substantial level of humic acids is produced thus enabling supply of carbon and nitrogen to soil microbes [285]. Despite the fact that nanofertilizers are a viable agricultural technology with generally favorable effects on soil microbiota, they may also potentially have detrimental repercussions. Rajput, [283] stated that there is a possibility that exposing soil to nanoparticles might be toxic to soil, resulting in a reduction in soil microbial biomass and enzyme activity which will eventually influence the microbial community structure. Research by Xu et al. [286] discovered that nanoparticles of TiO_2_ and CuO reduced microbial and enzymatic activity which effects the microbial composition in submergence paddy soil. Other findings revealed that ZnO, TiO_2,_ CeO_2_ and Fe_3_O_4_ decreased the soil enzymatic activity including, catalase, invertase, phosphatase and urease, thus, changing the population of soil microbial population [287]. The impact of nanoparticles on agriculture includes a variety of advantages and disadvantages. Yet, growing use of nanoparticles may endanger beneficial microbial populations as well as the soil and crops. Therefore, this technology has to be further fine-tuned to have beneficial impacts on soil microbiome.

### 7.2. Pesticides

Herbicides, insecticides, and fungicides are the three primary types of crop protection agents. The primary goal is to keep weeds, pest infestations, and disease under control. Pesticides come in a variety of forms, each of which is designed to combat certain pests. Pesticides are used in both conventional and organic farming, albeit the two systems are regulated differently. Organic farming permits only natural pesticides to be used, whereas conventional farming allows the use of synthetically generated pesticides [219]. Monocultures with extremely short rotations, in particular, results in significant pest pressure, necessitating the use of pesticides to maintain yields [288]. Pesticides that enter the soil in large concentrations have a direct impact on soil microbes. The organic pesticides are still generally believed to be much less environmentally hazardous compared to synthetic pesticides, despite the findings of multiple studies to the contrary [289,290]. 

Herbicides reduce the overall microbial abundance between 7 to 30 days following treatment, regardless of the kind of herbicide employed [291]. These negatively impact microbial biodiversity by affecting physiological or biosynthetic pathways [292]. Santos [293], found that within 12 days of applying fomesafen herbicide and mixes of fomesafen + fluazifop-p-butyl treatment, soil microbial biomass carbon (MBC) and mycorrhizal colonization decreased under the conventional till (CT) system. Hussain et al. [294] however, discovered that the interaction of herbicides with other substances is more sensitive to microbial populations compared to the usage of a singular herbicide, as seen in this study where butachlor and cadmium were employed in tandem. Other herbicides, when combined with heavy metals and inorganic fertilizers, decreased soil microbial functions [295,296]. Herbicides individually or in combination may disrupt the symbiotic relationship involving rhizobacteria and plants (legumes), preventing critical N_2_-fixation activities [297,298,299,300]. Herbicide application on legumes can alter nodulation and, consequently biological nitrogen fixation (BNF) through disrupting the infestation of rhizobacterial or disrupting the plant’s root tissues where infestation and node production takes place. They would also have an impact on *Rhizobium*’s phytochemical signalling, which is required for the coordination and control of BNF’s critical functions [294]. Triazines (bentazone, simazine, terbutryn and prometryn) that are extensively used could decrease rhizobial activity at doses higher than the acceptable rate [297]. At the prescribed field rates, fluazifop-butyl, sethoxydim, metolachlor and alachlor herbicides showed no negative impact on soybean yields and its BNF [301]. Glyphosate and paraquat (formulation containing ethylamine) [302] are non-selective herbicides that have been shown to inhibit N_2_-fixation in soybeans. 

Organophosphate insecticides (malathion, quinalphos, diazinon, dimethoate, chlorpyrifos and methyhpyrimifos) emanating from plant-to-soil runoff, produced a variety of impacts on the soil, including alterations on the fungal and bacterial populations [303]. Chlorpyrifos and methylpyrimifos are two of organosphosporus insecticides that are commonly used in farming [304]. High concentrations of methylpyrimifos (100–300 µg $g^{-1})$ or chlorpyrimifos (10–300 µg $g^{-1})$ can significantly reduce aerobic fixation, N_2_-fixing bacteria thus leading to decrease in N_2_-fixation in soil [305]. Same reduction in soil nitrification was observed when Fenamiphos was applied as this compound affects urease and dehydrogenase activities [306]. Diflubenzuron, another widespread insecticide used in agriculture stimulates the growth of N_2_-fixing bacteria in soil [307]. Metamidophos, like the other chemicals mentioned earlier, reduced microbial biomass by 41–83 percent compared to control [308]. This chemical however had the potential to significantly stimulate fungal populations [309].

While fungicides are used to combat fungal infections, it may also negatively impact beneficial fungi in the soil [279]. Fungicide has a direct impact on basic fungal life functions such as respiration, cell division and biosynthesis of sterol [310]. Fungicides usage on a regular basis result in detrimental impact on soil beneficial microorganism and its biochemical processes [311]. The detrimental consequences of fungicides on soil can be seen from the shifts in the biodiversity and abundance of microbial populations, as well as decrease in soil enzymes activities [312]. Baćmaga, Wyszkowska and Kucharski [312] reported on the effect of fungicides Falcon 460 EC on the microbial diversity and enzyme activity of the microbiome. The active ingredient of this fungicides includes, sproxamines, tebuconazole and tridimenol which have the ability to suppress sterol synthesis and interfere with membrane function [313,314]. 

Fungicides are also able to affect catalase, dehydrogenases, urease, acid and alkaline phosphatase activity, thus impacting the biodiversity of microbial groups such as *Bacillus*, *Rhizopus* and *Penicillium* [312]. Two fungicides, azoxystrobin and chlorothalonil, were recently demonstrated to have an effect as biological control agents which were utilized to prevent Fusarium wilt [315], demonstrating possible contraindications between biological controls and chemical pesticides. Apart from that, a study also found that the treatment with fungicides fenpropimorph can lead to the selection in populations of fenpropimorph-tolerant fungi [316]. The fungicide fenpropimorph reduces the proliferation of productive fungi in the first 10 days, while on the 17th day to 56th day, fenomimorph can drastically reduce the multitude of total culturable bacteria. A study on iprodione discovered that the community of soil bacteria will adapt faster and return to the original status faster at lower temperature with lower iprodione concentration compared to higher temperatures and higher iprodione concentrations [317]. Margarey and Bull [318] reported a significant reduction in total *Pseudomonas*, Actinobacteria and fungi due to the application of mancozeb, while an increase was observed in other bacterial population. This increase in certain bacterial population may be due to reduced competition as a consequence from the reduction of *Pseudomonas*, Actinobacteria and fungi in the soil. 

Plants are also protected by a variety of antimicrobials, nematicides, and hormones [279]. The antimicrobials tylosin, sulfachloropyridazine, and oxytetracycline decreased the Gram-positive bacterial colonies and hindered respiration of microbial communities [319]. This is consistent with alterations in the structure of microbial community following addition of tylosin in Westergaard et al.’s study [320]. Antibiotic alter the activity of enzymes and alter the ability of soil microorganisms to metabolize various carbon sources. It also has the ability to modify the community structure of various groups in the rhizosphere and shifts the total microbial biomass [321]. Nematicides are improbable to have an effect on the population of soil microbes, however the interaction may cause intermittent disruption in the functioning of food web in soil and result in short-term volatility of nematodes [303]. As discussed before, microbiota diversity and composition vary according to plant and soil makeup. This suggests that the microbial communities in these makeups are dynamic and influenced by the surrounding as well as plant-microbe and inter-microbial interactions. Therefore, the application of hormones auxin, gibberellins, cytokinin, ethylene and abscisic acids affect plant development such as shoot, root, and cell elongation which alters the formation of plant microbiome since root exudates such as organic acid, sugars and antimicrobial compounds will attract different group of microbes and alter the rhizosphere.

### 7.3. Irrigation Water

Water is a critical resource in agriculture for producing long-term plant yield. Water deficiency can have a negative impact on plant growth and physiological systems, resulting in negative results [322]. Water applications are numerous, but the most common is irrigation, livestock maintenance, and pesticide and fertilizer application. Irrigated agriculture has long been a key technique for increasing food production efficiency, and it is unquestionably the largest consumer of freshwater, causing a number of issues with water resources, including wastefulness of up to 50% in agriculture, and pollution due to a lack of basic sanitation and surface run-offs that carries dissolved loads of agricultural inputs such as fertilizers and pesticides [323]. In order to protect freshwater supplies, treated wastewater is introduced as an alternate source. There are multiple sorts of treated wastewater that originate from various sources such as aquaculture wastewater, saline wastewater, municipal wastewater and so forth. 

Generally, it is presumed that irrigation with treated wastewater would have an effect on functional diversity of soil microbes and its community structure and soil landscape. However, after extensive research, there is discrepancy with this statement. Study by Speir [324] described that, the use of treated wastewater improves plant development and increases soil biochemical activity, as measured by basal respiration and its relationship to soil microbial biomass, or metabolic quotient (qCO_2_), and the activity of several hydrolytic enzymes. Treated wastewater may also increase abundance of soil microbes, specifically fungal and bacterial community compositions [325], and change the ammonia-oxidizing bacteria in soils [326,327]. The significant microbiological threats that were related to the implementation of wastewater irrigation in agriculture are specific towards disruption of soil’s native microbial populations and the impact on their functional process [328]. 

Aquaculture wastewater can be another beneficial alternative source of water for agriculture. Chen’s [329] discovered that soils irrigated with aquaculture wastewater have greater diversity of bacterial communities. Treated soil contained numerous members of phyla Acidobacteria, Actinobacteria, Gemmatimonadetes, Chloroflexi, and few members of Plantomycetes, Proteobacteria and Bacteriodetes. The changes of chlorine (Cl) concentration in soil contributed to the changes in the composition of the bacterial community [329]. However, this study [329] observed that, the microbial functional diversity is lower in aquaculture wastewater treatment even though the soil has greater bacterial community richness and diversity. This finding suggests that the soil microbes in aquaculture wastewater treatment are functionally deficient despite their significant level of taxonomic diversity. The plausible reason for this effect is a transition from specialist (possessing specific gene functional) to generalist species (possessing functional genes that are expressed by many species) [329]. Evidently, increasing taxonomic diversity in generalist species would not enhance functional diversity because nearly all species share roughly identical genes. Contrastingly, greater taxonomical diversity of specialist gene will lead to increased functional diversity as every species has its own array of functional genes [330].

In mangrove soils that had been watered with saline wastewater, researchers discovered a decrease in microbial activity [331]. On the other hand, soil irrigated with municipal wastewater decreased the catalase activity at higher irrigation dosage [332]. Another study discovered a reduction in enzyme activity of β-glucosidase, alkaline phosphatase, urease, dehydrogenase and aryl sulfatase in wastewater-irrigated soils of agriculture [333]. This is in contrast to the Truu, Truu and Heinsoo [334] findings, which found a substantial rise in alkaline phosphatase enzyme in soils when treated with municipal wastewater irrigation. The increase of various enzyme activities in soils irrigated with municipal wastewater were also found in a report by Chen [335]. 

**Figure 2:**
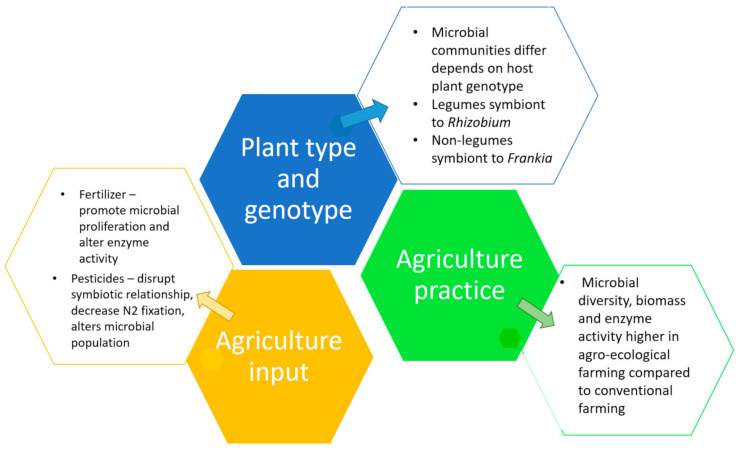
The connection between plant genotypes, agricultural practices and input on the soil microbial diversity.

The groundwater and wastewater influence on the soil microbiota might be both beneficial and destructive. To retain the soil microbial population in the rhizosphere, it is critical to determine the appropriate water content, whether beneficial or destructive, so that it does not harm the rhizosphere. On the other hand, additional research is needed to determine the optimum alternative for freshwater and improved comprehension of wastewater treatment in order to minimize water scarcity while somehow benefiting the soil microbiota and agricultural practices. Figure 2 provides a simplistic connection between the plant genotypes, agricultural inputs and practices on the soils microbial structure and diversity.

## 8. Future Prospect

An increased recognition of the intricate interplay between plants and microorganisms would pave the way for crop production to be more resilient to varied abiotic stresses by leveraging the rhizosphere microbiome. Abiotic stress has a negative influence on the soil microbiome, but soil microorganisms are able to resist such stressors by implementing several mechanisms to ensure their survival in the soil and to retain soil fertility in excellent condition for plant development. In drought stress, microbes can produce osmolytes and organic compounds [89,336], while in submergence stress, microbes can adjust their intracellular osmolarity and enhance cell wall stability [30]. Likewise in metal toxicity. microbes strategize to employ biotransformation, utilization of enzymes, extrusion, synthesis of exopolysaccharides (EPS), and metallothionein production, while in salinity, salt tolerant microbes may produce osmolytes. 

While many studies have been conducted under different stressors, different farming conditions and plant genotypes and environmental conditions; yet the information is varied depending on soil type and the degree of stress experienced. The entire microbe-microbe, plant microbe and microbe environment interaction are complicated and complex and still has many gaps that requires further study. Further, in most reports only one of the parameters or stressors has been studied individually and therefore the collective effect of the above-mentioned conditions on the soil microbiome is still lacking. In addition, studies should also be conducted to identify microorganisms that are effective against a host of biotic and abiotic stresses so that these microorganisms can be used as a consortium to improve plant resistance, growth, development and productivity. However, the identification of individual microbes or mixed cultures for field application will require extensive study before yielding good candidates for use as soil amendments, biofertilizers, or biocontrols. 

The influence of root exudates on the microbial community is indisputable, leading to the conclusion that various plant types and genotypes may result in distinct microbial populations based on these chemicals recruiting specific populations while inhibiting others. Apart from that, the soil environment plays a role in the formation of the rhizosphere, and the delicate interplay between the two must be well comprehended. The soil type and host genotype cooperatively regulate functions underlying the necessity of taking soil condition and plant genetic diversity into account when developing and applying synthetic microbiomes in the future. Further, the role of microorganisms in the development of root architecture, plant growth, and defense mechanism requires further study to completely understand the array of processes and genes turned on in response to systemic acquired resistance (SAR) and induced systemic resistance (ISR) in plants.

The soil microbiome is profoundly affected by agricultural practices and agricultural inputs. Using organic agricultural inputs and practicing agro-ecological farming techniques are undoubtedly beneficial to rhizospheric organisms while conventional farming, as well as the use of chemical agricultural inputs may adversely affect the soil microbiome. However, there is still a heavy dependence on conventional farming and chemical agricultural substances such as mineral fertilizers and pesticides since the end result is quicker and more promising and less expensive in terms of providing excellent crop production and stressor mitigation. Despite the fact that many studies have been conducted to identify alternatives to mineral fertilizers and pesticides, their utilization is still limited and their use is still not convincing. The interaction between pesticides composition and microbes that play a role in soil fertility are hard to predict. Therefore, more in-depth studies are required, as are alternative chemical fertilizers and pesticides that do not hamper the biodiversity of the soil.

In the past, culture-dependent techniques are used to study microbial communities, however most microbes in the environment are not culturable. The traditional microbiology methods are not robust enough to cultivate diverse microorganisms at the same time, plus the procedure is laborious and prone to contamination. The introduction of large parallel sequencing technologies has allowed researchers to investigate potentially beneficial microbial activities in entire communities, allowing us to study various aspect of plant-microbe and above and below ground microbial communities. In the recent decades, alternative green farming techniques have been explored, where microbes make an important constituent in this practice. Importantly, sustainable agriculture methods rely heavily on plant–microbiome interplay. Extensive research detailing how the plant immune response effects the microbiomes and how microbiomes aid plants in such processes is needed to have a better grasp of the possibilities for enhancing agricultural production and protection in the complex microbial ecosystems. With more research, it is anticipated that the influence of climate change and biodiversity on agriculture may be better understood in the future. 

## Figures and Tables

**Table 1:** The role of soil microbes in promoting growth in cultivated plants under abiotic stresses.

Microbes	Role	Plant	Stress
*Pseudomonas* sp.	Increase shoot biomass Increase flower number	- *Petunia hybrida* - *Impatiens wallerina* - *Viola wittrockiana*	Drought Nutrient [18]
	Improve germination rate	- *Arabidopsis thaliana* - *Gossypium hirsutum*	Salinity [19,20]
	Increase biomass Alter ABA and IAA content Improve antioxidant enzymes activity	*Lycopersicum esculentum*	Drought [21]
*Trichoderma* sp.	Produce IAA, phenols, and flavonoids Increase chlorophyll content Improve development rate	Wheat (*Triticum aestivum*)	Submergence [22]
	Increase seed biomass Increase tolerance toward salinity and drought stress	*Brassica napus*	Salinity Drought [23]
	Increase stomatal conductance Increase shoot dry weight Increase N and P uptake	Tomato (*Solanum lycopersicum)*	Drought [24]
	Improve S uptake Increase chlorophyll content Improve sucrose and sugar content in drought stress	Sugarcane (*Saccharum officinarum*)	Drought [25]
*Rhizobium* sp.	Accumulate more proline, soluble sugar, and protein Protect membrane system	*Medicago sativa*	Low Temperature [26]
*Bacillus* sp.	Increase proline accumulation Increase antioxidant enzyme activities Increase chlorophyll content Increase carotenoid content Prevent cell membrane damage	Tomato (*Solanum lycopersicum)*	Salinity [27]
	Improve growth development Increase chlorophyll and carotenoid content Increase salicylic acid content in both with and without stress Increase proline content Secrete IAA, and ACC under stress	*Capsicum annuum* cv. Geumsugangsan	Salinity Heavy Metal Drought [28]
